# Network diffusion modeling predicts spatiotemporal gray matter alterations in internet gaming disorder

**DOI:** 10.1017/S0033291726104462

**Published:** 2026-05-14

**Authors:** Shaoyu Cui, Min Wang, Xuefeng Xu, Meiting Wei, Xin Luo, Xuzhou Li, Zhitao Huang, Ouwen Huang, Guang-Heng Dong

**Affiliations:** 1 Yunnan Normal University, Kunming, China; 2 Ningbo University, Ningbo, China

**Keywords:** Internet gaming disorder, Network diffusion model, Gray matter volume, Brain connectome, Disease progression, Gaming craving, Reward system, Structural MRI, Neuropathological spread, Disease epicenter, Subcortical gray matter, Cognitive control

## Abstract

**Background:**

Recent research indicates that neuropathological alterations may propagate via brain networks, as illustrated by network diffusion models (NDMs). The application of NDM to internet gaming disorder (IGD) has yet to be evaluated. This study was set to identify possible epicenters of neuroanatomical alterations using NDM in IGD.

**Methods:**

Structural magnetic resonance imaging (MRI) data were obtained from 288 IGD participants and 165 matched recreational game users. Gray matter volume (GMV) was computed through CAT12 and segmented according to the Brainnetome Atlas. NMD was utilized to simulate the propagation of pathology. We initiated diffusion from each location to pinpoint probable epicenters of GMV alterations in IGD and correlated eigenmodes of the Laplacian matrix with observed atrophy and expansion patterns.

**Results:**

Abnormal brain regions with altered GMV were observed in IGD. Specifically, IGD demonstrated a great loss in GMV in the caudal cuneus gyrus, precentral and postcentral gyrus, as well as the cingulate cortex while simultaneously exhibiting an increase in the amygdala. The pallidus and putamen showed positive correlations with gaming craving. Both the right cingulate gyrus and the left amygdala were identified by the model as significant epicenters of disease dissemination.

**Conclusions:**

The results suggest that gray matter morphological abnormalities can predict temporal sequencing of pathology progression in IGD. Subcortical gray matter volume increases in reward-processing-related regions were positively correlated with gaming craving severity in IGD, consistent with altered reward processing and motivational drive in addiction models.

## Introduction

Internet gaming disorder (IGD) is characterized as a psychiatric disease marked by an inability to regulate excessive and disruptive gaming behaviors (Dong, Li, Wang, & Potenza, 2017; Wang et al., 2023). As a condition for more research, IGD was added to Section III of the DSM-5 in 2013 (*The Diagnostic and Statistical Manual of Mental Disorders*, Fifth Edition) (American Psychiatric Association [APA], 2013). The eleventh revision of the International Classification of Diseases (ICD-11) also categorizes gaming disease as a condition resulting from addictive behaviors (Jo et al., 2019). A recent meta-analysis indicated that the incidence of internet gaming disorder (IGD) is notably high (9.9%) among teenagers and young adults, constituting a substantial public issue, including vocational, social, and academic impairments (Gao, Wang, & Dong, 2022).

Various studies suggest that individuals with IGD exhibit varying degrees of structural abnormalities in the brain. A study showed that compared with the healthy controls, IGD participants showed significant lower gray matter density in the bilateral inferior frontal gyrus, left cingulate gyrus, insula, right precuneus, and right hippocampus (Lin, Dong, Wang, & Du, 2015). Yuan et al. observed reductions in brain gray matter volume (GMV) in the bilateral dorsal lateral prefrontal cortex, supplementary motor area, orbitofrontal cortex, cerebellum, and left rostral anterior cingulate cortex (Yuan et al., 2011). Another article also showed that compared with the recreational game user (RGU) group, the IGD group showed significantly decreased cortical thickness in the left lateral orbitofrontal cortex, inferior parietal lobule, bilateral cuneus, precentral gyrus, and right middle temporal gyrus. Moreover, significantly reduced cortical volume was observed in the left superior temporal gyrus and right supramarginal gyrus in the IGD group (Wang et al., 2018b).

Although previous structural functional magnetic resonance imaging studies have identified changes in GMV, density, and cortical structure among individuals with IGD (Niu et al., 2024). Recent evidence suggests that structural brain abnormalities are not randomly distributed. Instead, they emerge from focal brain regions (‘epicenters’) and spread to other areas, following the architecture of healthy brain networks in neuropsychiatric disorders such as Alzheimer’s disease, Parkinson’s disease, and schizophrenia (Shafiei et al., 2020; Wannan et al., 2019; Yau et al., 2018; Zhou et al., 2012). Neuroimaging studies reveal that structural brain abnormalities caused by clinical disturbances originate in focal epicenters and subsequently spread to unaffected brain regions, adhering to the intact structure and functional network (Brown et al., 2019; Yau et al., 2018; Zeighami et al., 2015; Zhou et al., 2012). These disease epicenters are typically brain regions characterized by dense interconnections, referred to as hubs, owing to their elevated metabolic demands and increased exposure to a toxic agent (Fornito, Zalesky, & Breakspear, 2015; Larivière et al., 2020).

To better understand this pathological spread, computational models have been created to elucidate how the configuration of the human connectome may promote the dissemination and aggregation of aberrant proteins in the brain. Raj et al. offer a model called the network diffusion model (NDM) to simulate problematic propagation (Shafiei et al., 2020). This model treats trans-neuronal pathology transmission as a diffusion process occurring within the healthy brain network, using abnormalities in gray matter as disease markers (Shafiei et al., 2020). In neurodegenerative diseases, the NDM was able to successfully capture the pathology’s trans-neuronal propagation and even forecast its future longitudinal progression (Raj et al., 2015; Shafiei et al., 2020). Research related to NDM encompasses not only neurodegenerative diseases but also psychiatric disorders (Chopra et al., 2023; Liu et al., 2024), making it theoretically applicable to IGD research. Notably, all these studies utilize GMV as an indicator. However, its applicability to IGD remains unexamined.

In this study, we explored whether NDM could predict the severity of neurodegeneration in IGD compared to controls. Following a previously established framework (Poudel, Harding, Egan, & Georgiou-Karistianis, 2019; Shafiei et al., 2020), we implemented NDM to assess whether network diffusion via the human brain connectome correlates with the degree of atrophy observed in IGD. We hypothesized that this model would effectively predict the severity of structural brain changes, as reflected by gray matter volume abnormalities in IGD. Additionally, we aimed to investigate the temporal progression of pathological changes originating from the initial seed region identified by the NDM (Liu et al., 2024).

## Methods

### Ethics

The Human Investigation Committee at Yunnan Normal University approved the study. Informed consent was acquired in writing from all participants. The complete experimental protocol adhered to the Declaration of Helsinki.

### Participants

In total, we recruited 453 participants (288 IGDs and 165 RGUs) through posters and online advertisements. All participants were right-handed with normal or corrected to normal visions. Additionally, the two groups were matched on sex and age (see Table 1). Before formal scanning, each participant completed a written informed consent form and underwent a structured psychiatric interview (Mini International Neuropsychiatric Interview) (Lecrubier et al., 1997). All participants were also free of psychiatric and neurologic disorders (e.g., major depression, anxiety disorders, schizophrenia, and substance dependence disorders). All participants were medication-free and were instructed not to use any substances, including coffee, on the day of scanning (Chen et al., 2024).

**Table 1.** Participant demographics

Characteristic	IGD	RGU	p
Sex	Male = 158, female = 92	Male = 130, female = 73	0.854
Age	24.14 ± 1.99	21.49 ± 2.53	0.125
IAT	67.42 ± 11.492	41.32 ± 10.38	0.000
DSM	6.51 ± 1.519	2.61 ± 1.378	0.000
Craving	55.50 ± 17.346	35.80 ± 15.346	0.000

*Note*: IAT, internet addiction test; DSM, Diagnostic and Statistical Manual of Mental Disorders-5; IGD, internet gaming disorder; RGU, recreational game user.

The criteria for screening addiction were derived from the Young’s online internet addiction test (IAT) and the nine diagnostic criteria for internet gaming disorder established by the DSM-5 Committee (American Psychiatric Association, 2013; Young, 2009). The criteria for inclusion in IGDs are as follows: 1) IAT score exceeding 50; 2) DSM-5 score surpassing 5; 3) online gaming as the predominant internet activity; 4) daily online gaming duration exceeding 2 hours and cumulative gaming experience exceeding 2 years. The RGU group engaged in online gaming but did not shift from nonaddiction to addiction (Kuss & Griffiths, 2012; Wang et al., 2017). Compared with the general control group, the RGUs had greater gaming experience and extended gaming duration. The requirements for inclusion in the RGUs were as follows: 1) IAT scores under 50 and DSM-5 scores less than 5; 2) hours of gaming indicative of IGD; and 3) absence of guilt regarding online gaming that disrupts daily life (e.g., academic, familial, occupational, social obligations).

### Magnetic resonance imaging data acquisition

The magnetic resonance imaging data were acquired using a Siemens Trio 3.0 T scanner (Siemens, Erlangen, Germany). The specific parameters used were as follows: repetition time (TR) = 2000 ms, 33 interleaved slices, echo time (TE) = 30 ms, thickness = 3.0 mm, flip angle = 90°, field of view (FOV) = 220 mm × 220 mm, and matrix = 64 × 64. Head motions were minimized by filling the empty space around the subjects’ heads with sponge and fixing their lower jaws with tape.

### MRI data preprocessing

We processed the 3D-T1 structural images of all subjects using the segment function in CAT12 (http://dbm.neuro.uni-jena.de/cat12/) within the MATLAB 2018a software. The ICBM space template was selected based on East Asian brains, while other options were applied according to the recommended procedures. The total intracranial volume (TIV) was recorded for the following analyses (Brown et al., 2019; Han et al., 2023).

### Heterogeneity of GMV and generation of structural connectome

First, the obtained gray matter maps were smoothed using a 6 mm full width at half maximum (FWHM) Gaussian kernel (Ashburner, 2009; Han, Yang, & Xu, 2021). Next, we extracted regional GMVs for both IGD and RGU groups based on the Human Brainnetome Atlas, which comprises 246 brain regions [BNA246] (Fan et al., 2016; Tzourio-Mazoyer et al., 2002). This yielded an M × N GMV matrix, where M represents the total number of subjects and N denotes the total number of brain regions. Subsequently, we applied a generalized linear model (fitglm, Matlab2018a), incorporating TIVs as a covariate, to obtain t-values (coefficient estimates) and p-values for all N brain regions (Asimit, Badescu, Chen, & Zhou, 2025). Multiple comparison correction was applied at the ROI level using the FDR method; regions with adjusted *p*-values <0.05 are presented in Table 2. The t-values were then used as input for the subsequent NDM analysis. The structural connectome utilized was derived by identifying all streamlines intersecting the seed from a whole-brain tractography template with roughly 12 million fibers. This template was created from a diffusion-weighted MRI dataset comprising 985 people supplied by the Human Connectome Project (http://www.humanconnectomeproject.org) (Elias et al., 2024; Glasser et al., 2013), adhering to methodologies employed in prior research (Germann et al., 2021; Li et al., 2020).

**Table 2.** Comparison of regional volumes in IGD and RGU

Brain regions	RGUs (cm^3^)	IGDs(cm^3^)	Difference ranges (cm^3^)	Confidence intervals	RGU vs IGD (estimates)	RGU vs IGD (*p*-values)	Cohen’s d
Middle Frontal Gyrus	3.957	3.829	[−3.683, 2.466]	(−0.229, −0.028)	−0.113	0.018	−0.25
Inferior Frontal Gyrus	1.510	1.562	[−1.231, 1.350]	(−0.099, −0.004)	−0.046	0.045	−0.21
Precentral Gyrus	3.230	3.049	[−2.898, 2.527]	(−0.272, −0.091)	−0.172	0.000	−0.39
Paracentral Lobule	1.264	1.294	[−0.767, 0.737]	(−0.058, −0.002)	−0.026	0.045	−0.20
Postcentral Gyrus	3.006	2.903	[−1.792, 1.805]	(−0.163, −0.044)	−0.097	0.002	−0.34
Insular Gyrus	1.327	1.289	[−0.782, 0.913]	(−0.065, −0.009)	−0.033	0.018	−0.25
Cingulate Gyrus	3.185	3.050	[−2.362, 2.053]	(−0.214, −0.056)	−0.123	0.002	−0.33
Pallidus	0.806	0.871	[−0.552, 0.600]	(0.044, 0.087)	0.068	0.000	0.59
Putamen	1.833	1.943	[−1.238, 1.321]	(0.062, 0.158)	0.115	0.000	0.44
Thalamus	1.037	1.170	[−0.754, 0.916]	(0.101, 0.165)	0.133	0.000	0.79
Amygdala	0.844	0.837	[−0.024, −0.01]	(−0.511, −0.500)	−0.005	0.540	−0.08

*Note*: MFG, middle frontal gyrus; IFG, inferior frontal gyrus; IGD, internet gaming disorder; RGU, recreational game user. Only the regions showing loss of volumes in IGD at *p* < 0.05 are shown in the table. All *p*-values denote FDR corrected.

### Network diffusion model

We used a network diffusion model (NDM) to empirically examine whether GMV loss and growth spreads through the brain via diffusion and whether specific brain regions function as sources or epicenters of this pathological dissemination (Chopra et al., 2023). The network diffusion model is used as described in Shafiei et al. (2020). A graph representing an undirected brain network can be expressed as **G** = ( 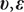 ), where 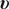 is the set of brain parcels (nodes) given by 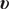 = ( 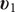 , 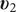 , …, 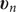 ) and 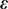 is the set of connections between 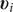 and 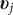 (edge) given by 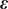 = ( 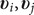 ). The network diffusion model treats the edge ( 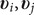 ) as a conduit that connects nodes 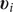 and 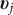 through which diffusion can occur (Poudel et al., 2019). According to the diffusion model, spread of pathology at time 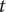 from region 1 (unaffected) to region 2 (affected) can be modeled as follows:
(1)

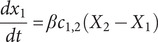

where 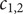 is represented as connection strength between regions 1 and 2, which is measured by fiber tractography (Behrens et al., 2007). β is the diffusivity constant controlling propagation speed (assumed to be 1 in our analyses). By incorporating spectral graph theory and extending the understanding of brain regions to the entire network, we derive the so-called network heat equation (Kondor & Lafferty, 2002).
(2)

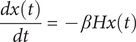

where 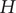 is the well-known symmetric normalized graph Laplacian.
(3)

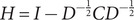

where D is the diagonal matrix with diagonal elements representing the degree of each node, defined as the total of weighted connections originating from the node. To account for regions with significantly varying out-degrees, we employed the degree-normalized form of the Laplacian matrix (Pandya et al., 2019; Raj et al., 2015). From matrix algebra, Equation 2 is satisfied by
(4)

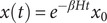

where 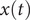 denotes the vector consisting of the amount of diffusion of pathology at node 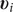 at time 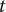 , beginning from an initial distribution of pathology given by 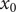 at time zero. Then, the diffusion process 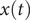 can be estimated as follows:
(5)

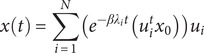

where 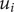 represents eigenvectors of the Laplacian matrix. Eigen vectors of the Laplacian matrix (eigenmodes) associated with smaller eigen values represent persistent modes of diffusion in the human brain connectome.

**Figure 1.**
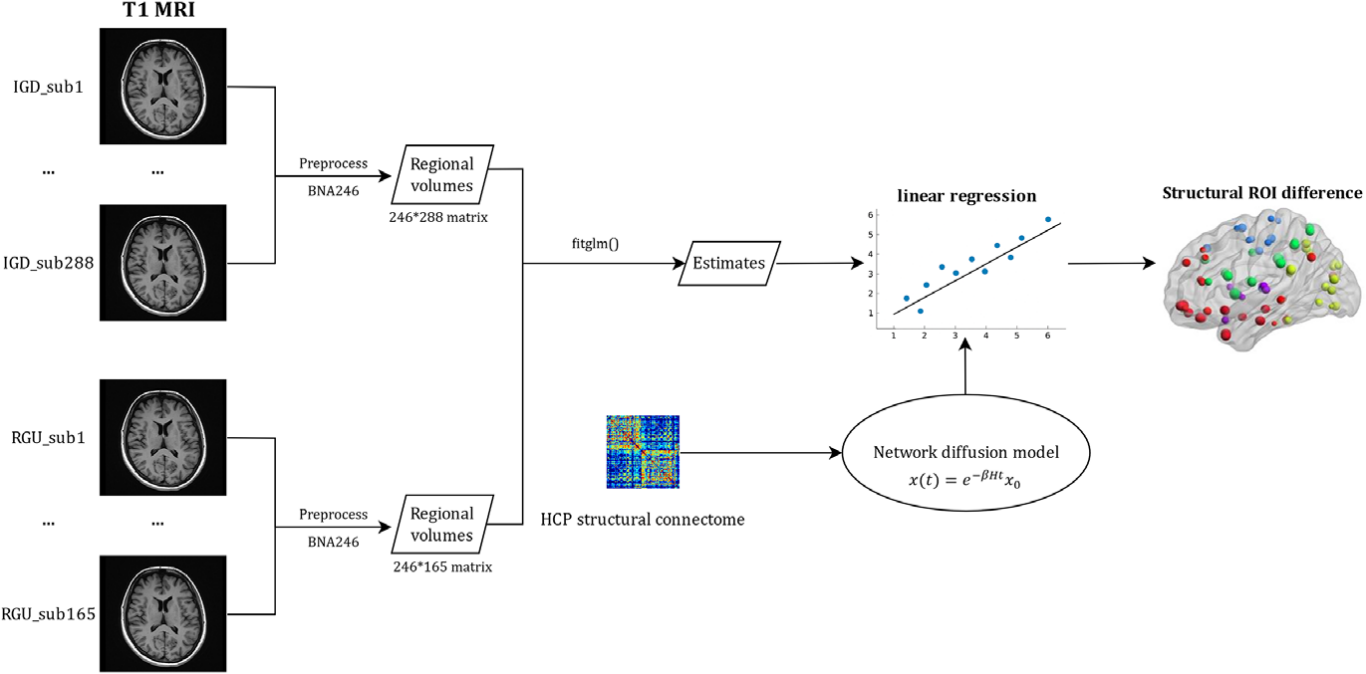
A flow diagram for the data analysis process used in the study. *Note*: Structural MRI analysis was performed on T1-weighted images from two cohorts: individuals with internet gaming disorder (IGD, N = 288) and regular game users (RGU, N = 165). Using CAT12 pipeline, all scans underwent preprocessing and were parcellated into 246 gray matter regions according to Human Brainnetome Atlas. Atrophy in these regions was estimated by calculating the estimates of the difference between IGD and RGU. Human Connectome Project (HCP) structural connectome was used to model network diffusion. In the first analysis, we used MATLAB’s fitglm function to calculate the estimates and *p*-values of all participants across 246 gray matter dimensions. In the second analysis, network diffusion was run on the HCP structural connectome by repeatedly initiating diffusion from each region of the brain. Finally, we compared the structural differences at different seed points.

To choose the eigenmode that best represented the atrophy and expansion pattern in IGD, the absolute values of the first five eigenvectors of the Laplacian matrix were correlated with the measured atrophy and expansion (*t*-values of the difference between controls and IGD) using Spearman’s correlation. Distinct associations were additionally conducted for cortical and subcortical areas. Any correlation with *p* < 0.01 (family-wise adjusted *p*-value criterion for the initial five eigenmodes utilized in the correlation) was deemed significant (see Figure 1).

### Repetitive network diffusion to identify the epicenters of disease

We employed the identical procedure as Liu (Poudel et al., 2019). We consistently initialized the model by designating each of the 246 regions as the starting seed, establishing the initial state to 1 for the seed region and 0 for all other regions. During each initialization, with a constant of 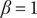 , the NDM was employed to estimate diffusion over all regions from time *t* = 0 to 50. This allowed us to ascertain if a diffusion process originating from each region produced a spatial distribution of volume loss that corresponded with the empirically observed patterns. To examine atrophic brain regions, we initially set all negative values in the estimates from fitglm to zero, followed by the use of a network diffusion model for subsequent analysis. To examine expansion brain regions, we reversed the fitglm estimates, set negative values to zero, and then calculated the findings using the identical network diffusion model.

### Randomized networks as ‘null’ distribution

To test whether the association between the NDM-predicted and observed GMV alterations is specific to the intrinsic architecture of the brain connectome, we compared the empirical model fits against a null distribution derived from degree-preserving randomized networks (Chopra et al., 2023; Poudel et al., 2019).

Using the null_model_und_sign function from the Brain Connectivity Toolbox (with default parameters), we generated 1,000 randomized networks by rewiring the original structural connectome while preserving the degree distribution of each node (Rubinov, Kötter, Hagmann, & Sporns, 2009). We then applied the 95th percentile method and calculated empirical p-values to determine whether the observed correlations significantly exceeded those expected by chance. Additionally, we plotted the distribution of maximum correlations between predicted and observed atrophy (and expansion) obtained from the randomized networks.

## Results

### Pattern of degeneration in IGD compared to controls

The brain regions showing volume loss in IGD compared to the RGU group (*p* < 0.05) are listed in Table 2. The regions most affected are in the precentral gyrus, postcentral gyrus, and cingulate gyrus showing greatest loss of volumes. In the middle frontal gyrus, inferior frontal gyrus, paracentral lobule, and insular gyrus also show volume loss in IGD. Concurrently, we observed an increase in GMV within the globus pallidus, putamen, and thalamus among IGD.

### The brain regions critical for generating the spatial distribution of atrophy

**Figure 2.**
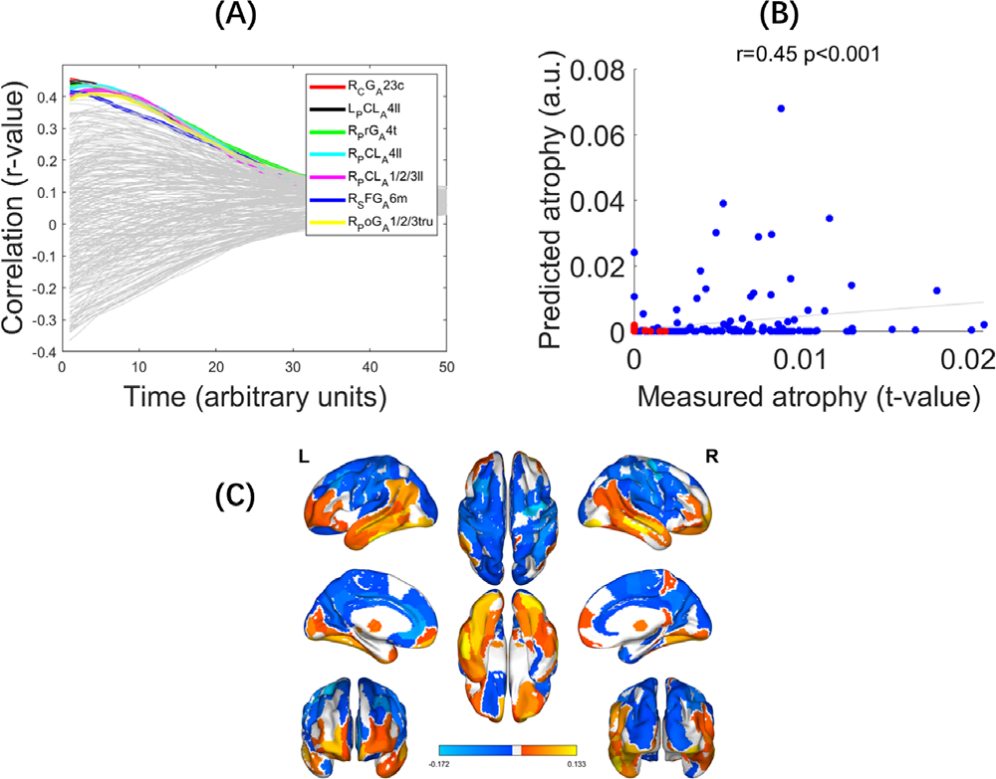
Spatial distribution of diffusion and atrophy over time. *Note*: (a) Curves showing evolution of correlation between the measured and predicted atrophy when the spread was initiated from each region in the Desikan‐Killiany atlas. Y‐axis shows the correlation values (Pearson correlation) and x‐axis shows time in number of years. The regions showing the highest correlation (Pearson correlation) between the measured (estimates of the difference between IGD and RGU) and predicted atrophy (amount of diffusion) are the right cingulate gyrus (red), bilateral paracentral lobule (black and blue and yellow), right precentral gyrus (green), right superior frontal gyrus (dark blue), and right postcentral gyrus (purple). (b) Scatter plot of the correlation between predicted and measured atrophy at the initiation of diffusion‐based spread from the cingulate gyrus. (c) Visual representation of structural ROI differences.

Figure 2 displays the results of repeated diffusion analysis across all 246 cortical–subcortical regions of the Desikan-Killiany atlas, modeling potential disease origins and spread through the healthy brain connectome. The cingulate gyrus, bilateral paracentral lobule, precentral gyrus, superior frontal gyrus, and postcentral gyrus exhibited strong to moderate associations between predicted and measured atrophy (Figure 2a,b). Figure 2c illustrates structural variations among regions of interest. The strongest association emerged when diffusion originated in the cingulate gyrus (*r* = 0.45, *p* < 0.001), corresponding to an explained variance of R^2^ = 0.203. To assess robustness, we further performed NDM analyses on IGD-related atrophy patterns using β = 0.8 and 1.2, t = 30 and 40. Across all tested parameter combinations, the right cingulate gyrus consistently yielded a correlation of r = 0.45, confirming the stability of this finding. Detailed procedures are available in Supplementary Material (Figure S1).

### The brain regions critical for generating the spatial distribution of expansion

**Figure 3.**
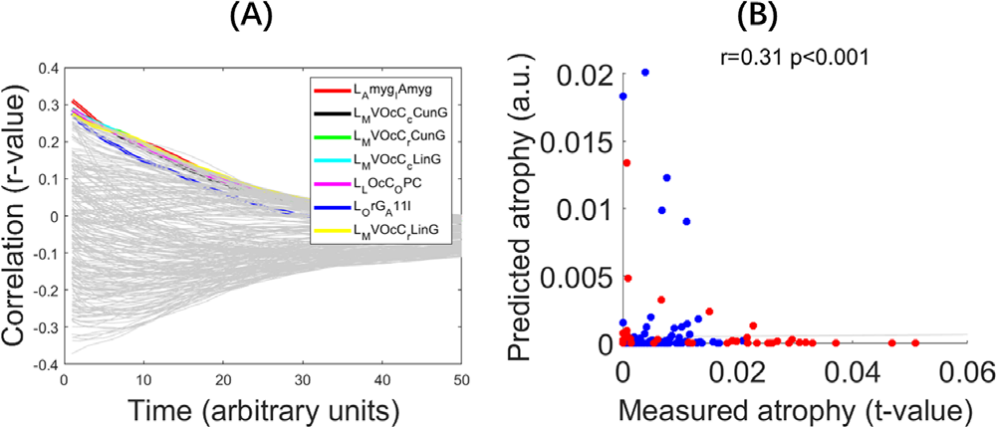
Spatial distribution of diffusion and expansion over time. *Note*: (a) Curves showing evolution of correlation between the measured and predicted expansion when the spread was initiated from each region in the Desikan‐Killiany atlas. Y‐axis shows the correlation values (Pearson correlation), and x‐axis shows time in number of years. The regions showing the highest correlation (Pearson correlation) between the measured (estimates of the difference between IGD and RGU) and predicted expansion (amount of diffusion) are the left lateral amygdala (red), left cuneus gyrus (black and green), left caudal and rostral lingual gyrus (blue and yellow), left occipital polar cortex (purple), and left lateral orbital gyrus (dark blue). (b) Scatter plot of the correlation between predicted and measured expansion at the initiation of diffusion‐based spread from the amygdala.

Mirroring the processing pipeline for atrophy mapping, the left amygdala, left caudal cuneus gyrus, left caudal and rostral lingual gyrus, left occipital polar cortex, and left lateral orbital gyrus demonstrated robust to intermediate associations between predicted and measured expansion (Figure 3a,b). The strongest association emerged when diffusion originated in the left amygdala (*r* = 0.31, *p* < 0.001), corresponding to an explained variance of R^2^ = 0.096. To assess the robustness of NDM analyses for expansion, we used the same parameters as for atrophy. The left amygdala consistently showed a correlation of r = 0.31 across all parameter combinations (β = 0.8, 1.2; t = 30, 40). Details are in Supplementary Material (Figure S2).

### Correlations analysis between GMV and Craving

**Figure 4.**
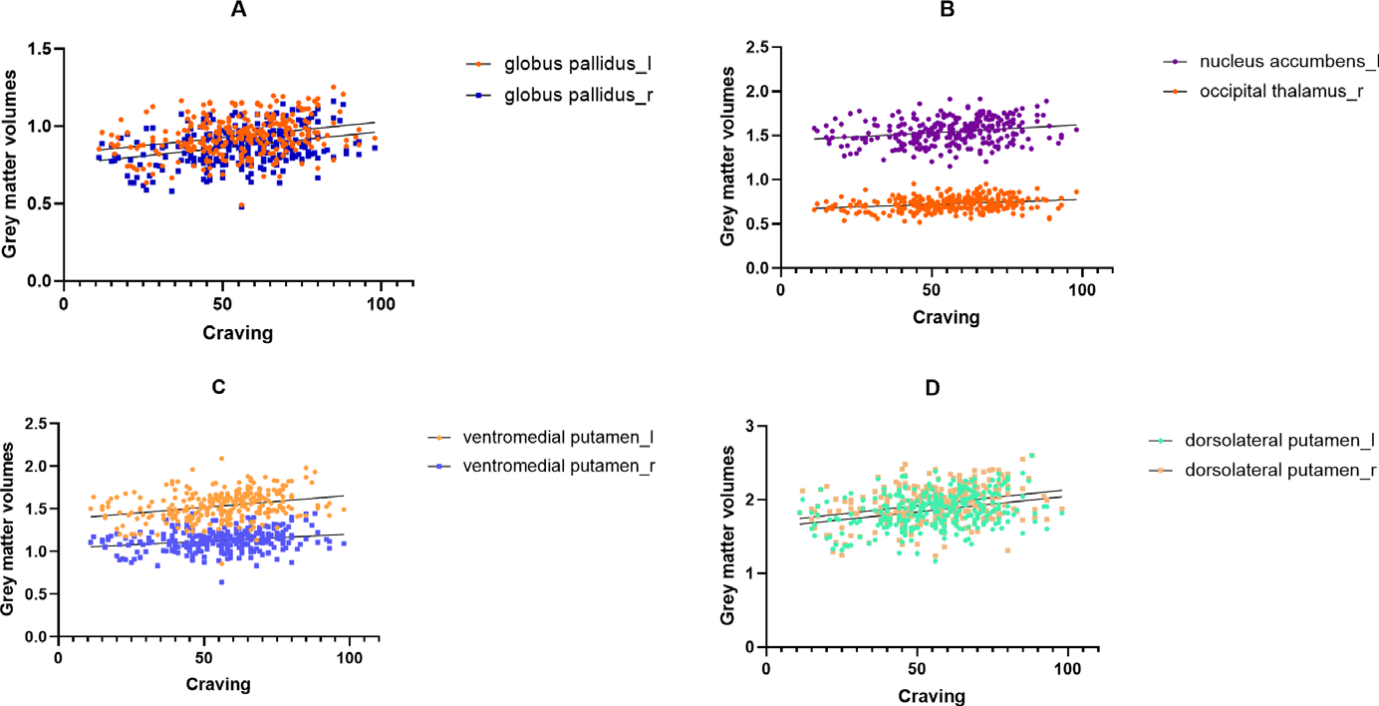
Correlation analysis between GMV and craving in IGD. *Note*: (a) Correlation results between game craving scores and the bilateral globus pallidus. (b) Correlation results between game craving scores and right occipital thalamus and the left nucleus accumbens. (c) Correlation results between game craving scores and the bilateral ventromedial putamen. (d) Correlation results between game craving scores and the bilateral dorsolateral putamen.

Following Bonferroni correction, game craving scores in the IGD group exhibited significant positive correlations with gray matter volume (GMV) in multiple brain regions. Specifically, GMV in the bilateral globus pallidus (left: *r* = 0.288, *p_bonf_* = 0.000; right: *r* = 0.310, *p_bonf_* = 0.000), bilateral ventromedial putamen (left: *r* = 0.292, *p_bonf_* = 0.000; right: *r* = 0.235, *p_bonf_* = 0.000), and bilateral dorsolateral putamen (left: *r* = 0.307, *p_bonf_* = 0.000; right: *r* = 0.307, *p_bonf_* = 0.000) showed positive associations with craving scores. Additionally, GMV in the left nucleus accumbens (*r* = 0.222, *p_bonf_* = 0.000) and right occipital thalamus (*r* = 0.241, *p_bonf_* = 0.000) was also positively correlated with game craving (see Figure 4).

### Null model validation

To assess the specificity of our NDM findings, we compared the empirical correlations against 1,000 degree-preserving randomized networks. For the atrophy epicenter (right cingulate gyrus), the empirical correlation (r = 0.453) significantly exceeded the 95th percentile of the null distribution (r = 0.107, p < 0.001). For the expansion epicenter (left amygdala), the empirical correlation (r = 0.309) was also significantly higher than the null 95th percentile (r = 0.112, p < 0.001). Detailed results are presented in Supplementary Figure S3.

## Discussion

Consistent with our hypothesis, the findings demonstrate that the trans-neuronal spread of pathology in IGD follows the brain’s structural connectome, effectively recapitulating the observed patterns of neurodegeneration and disconnection. In comparison to controls, individuals with IGD demonstrated atrophy in the precentral, postcentral, and cingulate gyri, alongside enlargement in the amygdala, whereas the pallidus and putamen revealed volume alterations associated with craving. Axonal disconnection was particularly evident when the right cingulate gyrus and left amygdala were designated as illness epicenters. The results indicate that network diffusion models may facilitate the prediction of IGD progression and offer a mechanistic framework for comprehending its neuropathological dissemination.

### Common abnormal GMV changes in IGD and RGU

As with prior neuroimaging findings, our study revealed significant gray matter volume (GMV) reductions in individuals with IGD compared to RGU controls, particularly in the cingulate gyrus, insula, middle frontal gyrus, and inferior frontal gyrus (Lin et al., 2015; Wang et al., 2015; Weinstein, Livny, & Weizman, 2017; Weng et al., 2013; Zhou et al., 2011). Notably, we extended these observations by identifying additional GMV decreases in the precentral gyrus, postcentral gyrus, and paracentral lobule, regions that have received less attention in previous IGD research.

The cingulated gyrus is the key part of the limbic system. This is in charge of controlling emotions, mood, motivation, and other emotional states (Mayberg, 1997). The anterior cingulated region has been implicated in motor control, cognition, and arousal/drive state (Goldstein et al., 2007; Paus, 2001). The posterior cingulated, which is also a part of a larger ‘default system’ of cortical areas, has been implicated in self-referential functions (Bush, Luu, & Posner, 2000). Insula is critical to the high-level cognitive control and reward processing (Menon & Uddin, 2010; Sharp et al., 2010). A previous study has shown that IGD participants showed significant lower gray matter density compared to controls in insula (Lin et al., 2015). The medial frontal gyrus, a region inside the prefrontal brain, is critical to cognitive control activities (MacDonald, Cohen, Stenger, & Carter, 2000; Yuan & Raz, 2014). The inferior frontal gyrus serves as a pivotal hub in the fronto-basal ganglia circuitry, facilitating attentional allocation, affective processing, inhibitory control, and the oversight and regulation of behavior (Moreno-López et al., 2012). Reduced gray matter density and gray matter volume have been found in both regions among individuals with IGD in previous studies (Choi et al., 2017; Wang et al., 2015). The precentral gyrus, postcentral gyrus, and paracentral lobule are considered key regions within the sensorimotor network, implicated in the regulation of bodily responses (Hanakawa, Dimyan, & Hallett, 2008; Wei et al., 2025), the integration of sensorimotor information, and the coordination of physical movement (Desmurget et al., 2014; Graziano, Charlotte, Taylor, & Moore, 2002). Although no relevant literature on gray matter volume was found, reductions in cortical thickness and gray matter density can still be found in structures similar to those in the literature (Sun et al., 2014; Wang et al., 2018a, 2018b). The observed structural abnormalities may suggest disrupted cognitive control, heightened reward sensitivity, and reduced sensitivity to loss in relevant brain regions.

### The right anterior cingulate cortex is the most probable epicenter of atrophy in IGD

This study examines the relationship between structural brain abnormalities and the normal structural brain connectome using NDM, which posits that pathology progresses trans-neuronally as a diffusion process. Seeding at the right anterior cingulate cortex (ACC) revealed a peak correlation between NDM predicted structural abnormalities and actual abnormalities, indicating that NDM effectively mirrors the patterns of observed structural anomalies and that gray matter morphological abnormalities are limited by the structural integrity of the healthy brain network in IGD. The NDM posits that the most probable epicenter of atrophy is the right anterior cingulate cortex in IGD (Goodkind et al., 2015).

The ACC serves as a central ‘hub’ in addiction-related neural networks of cognitive functions, including, but not limited to, decision-making, cognitive inhibition, emotion, and motivation (Zhao et al., 2021). One research found that IGD showed decreased glucose metabolism in right ACC compared to that in the HC (Kim et al., 2019). Another transcranial direct current stimulation (tDCS) research observed significant differences in the resting-state functional connectivity changes from the right dorsolateral prefrontal cortex between the post-tDCS and pre-tDCS states in the ACC among IGD (Jeong et al., 2024). Therefore, the NDM seeding at the right ACC may not only recapitulate the patterns of gray matter morphological atrophy and reveal the temporal sequencing of pathological progression in IGD but also lay a foundation for clarifying the causal role of this Epicenter in IGD pathogenesis. The right cingulate gyrus-based NDM explained 20.3% of GMV atrophy variance, supporting its role as a key epicenter in IGD-related structural alterations. Specifically, this NDM-derived atrophy pattern helps link the ACC’s structural changes to IGD-related abnormal brain activity, providing initial evidence for its potential as a diagnostic biomarker and a target for precise interventions.

### The left amygdala is the most probable epicenter of expansion in IGD

Seeding at the left amygdala also revealed a peak correlation between NDM predicted structural abnormalities and actual abnormalities, indicating that the most probable epicenter of expansion is the left amygdala in IGD. The amygdala is considered one of the key brain regions in the reward system (Shi, Zhao, Zhou, & Shi, 2025).

A study found that individuals with IGD exhibited significantly larger bilateral amygdala GMV compared to healthy controls (Yoon et al., 2017). However, a recent meta-analysis studies in IGD reported convergent GMV reductions in the right putamen (breakdown to the amygdala in 136 voxels) (Qin et al., 2020). These conflicting findings may stem from differences in sample size (19 IGD participants versus 237 GD participants) and voxel size (voxels >800 versus 136 voxels). Although previous findings on amygdala alterations have been inconsistent, network diffusion modeling revealed that the amygdala showed the strongest correlation between predicted and measured expansion across brain regions. One reasonable proposal is individuals with IGD may experience amygdala hypertrophy in the early stages, which then shifts to atrophy over the long term. The left amygdala-based NDM explained 9.6% of GMV expansion variance, further supporting its role as a key epicenter in IGD-related structural changes. Given these findings, future studies should employ NDM to systematically characterize the neuroprogressive patterns of expansion in IGD with a focus on validating whether the expansion-related Epicenters can serve as prognostic indicators for treatment response. Detailed investigations of amygdala gray matter morphology should be paired with developing GMV-based quantitative models. Such models could aid clinical diagnosis and severity assessment (by linking GMV changes to IGD symptom intensity), bridging structural findings with practical clinical tools.

### Craving rises with subcortical gray matter volume

In this study, the GMV in bilateral globus pallidus, ventromedial putamen, and dorsolateral putamen demonstrated significant positive correlations with craving scores. Notably, structural variations in the left nucleus accumbens and right occipital thalamus were also independently associated with game craving scores. Consistent with the characterization by Yeo et al. (Thomas Yeo et al., 2011), these ROIs belong to subcortical networks (SCN), which recent studies have identified as the network most associated with addiction (Bell & Shine, 2016). The SCN is responsible for reward processing (Heilig, Augier, Pfarr, & Sommer, 2019; Zhou et al., 2022). A recent study has demonstrated positive correlations between the Intra-Modular participation coefficient in SCN and gaming craving in IGD (Ma et al., 2024). These findings indicate that greater IGD severity is associated with increased subcortical GMV. Theoretical models of IGD suggest that diminished executive control over strong motivational impulses, such as cravings triggered by gaming cues, may hinder individuals with IGD from discontinuing gaming despite negative repercussions (Brand et al., 2019; Dong & Potenza, 2014), aligning with our findings.

## Limitations

Although this study provides novel insights into IGD’s neuropathology through network diffusion modeling, several limitations should be noted. First, NDM model assumed linear pathology progression; future studies could explore nonlinear diffusion patterns. Second, the cross-sectional design precludes causal inferences, necessitating longitudinal investigations to validate the observed progression patterns. Third, we focused exclusively on structural abnormalities, incorporating that functional connectivity data could provide a more comprehensive understanding. Forth, despite the large sample size in our study, discrepancies between groups still existed. In future studies, stricter control over sample characteristics and expansion of sample diversity (different IGD subtypes) should be implemented to enhance the specificity and sensitivity of these specific regional abnormalities. Fifth, the current study adopted a cross-sectional design. Future studies could use longitudinal data (12–24 months) to confirm our findings. Sixth, our use of template structural connectomes instead of patient-specific ones is also a limitation that merits future exploration.

## Conclusion

This study illustrates that network diffusion modeling accurately represents the trans-neuronal diffusion of neuropathological alterations in IGD, indicating the right anterior cingulate cortex and left amygdala as principal epicenters for atrophy and expansion, respectively. Structural anomalies in reward-related subcortical areas (globus pallidus, putamen, nucleus accumbens) were associated with the severity of cravings, reinforcing addiction models that posit weak executive control over cue-induced impulses. Our research establishes a framework for comprehending the neuroprogressive characteristics of IGD via connectome-based degeneration patterns, integrating behavioral addiction mechanisms with principles of network neuroscience. This methodology provides novel pathways for forecasting IGD advancement and formulating targeted strategies for IGD ailments.

## Supporting information

10.1017/S0033291726104462.sm001 Cui et al. supplementary material Cui et al. supplementary material

## Data Availability

All data supporting this study are presented in the text and in the Supplementary material. The raw dataset is not available for public download, but access requests can be made to researchers.

## Supplementary material

The supplementary material for this article can be found at http://doi.org/10.1017/S0033291726104462.
