# Supplementary material for: Network diffusion modeling predicts spatiotemporal gray matter alterations in internet gaming disorder
Source: Psychol Med. 2026 May 14;56:e150. doi: 10.1017/S0033291726104462 (PMC13200156; doi:10.1017/S0033291726104462)
Supplement: Cui et al. supplementary material [file S0033291726104462sup001.docx]

Supplementary materials for:

# **Network Diffusion Modeling Predicts Spatiotemporal Gray Matter Alterations in Internet Gaming Disorder**


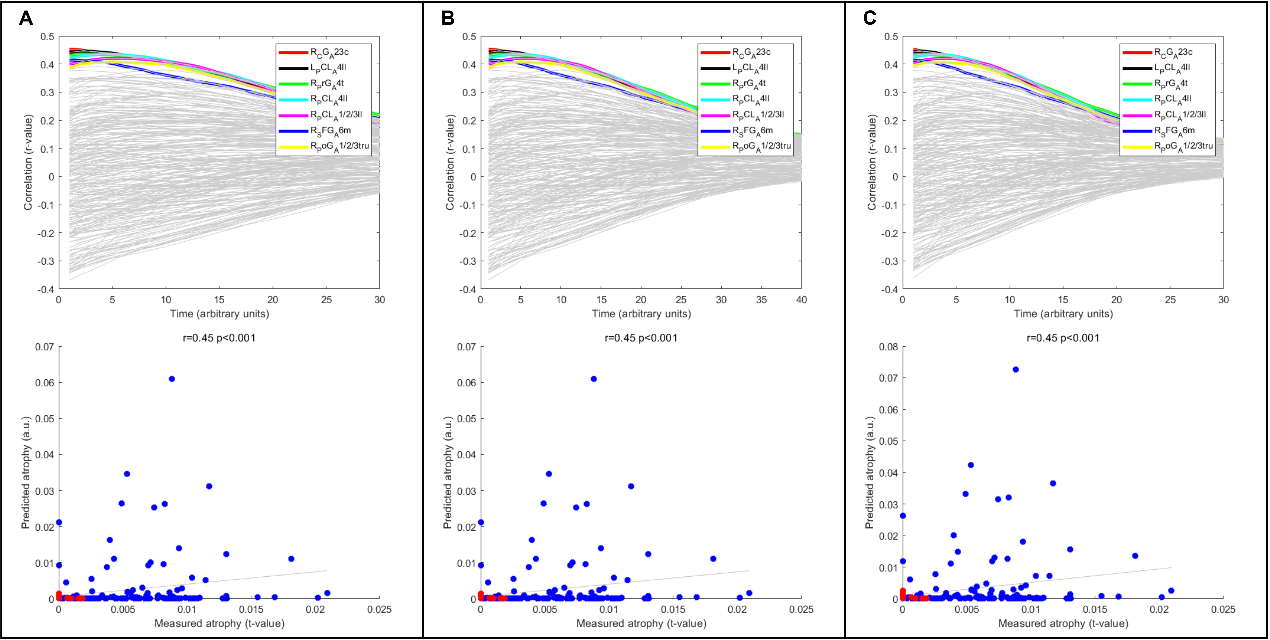


**Figure S 1. Robustness analysis of atrophy through NDM.**

**Figure legend:** (A), (B) and (C) show the results of predicting the atrophy epicenter of IGD using NDM with different combinations of β and t. (A) β = 0.8, t = 30; (B) β = 0.8, t = 40; (C) β = 1.2, t = 30. The regions showing the highest correlation between the measured and predicted atrophy are the right cingulate gyrus (red), bilateral paracentral lobule (black and blue and yellow), right precentral gyrus (green), right superior frontal gyrus (dark blue), and right postcentral gyrus (purple).


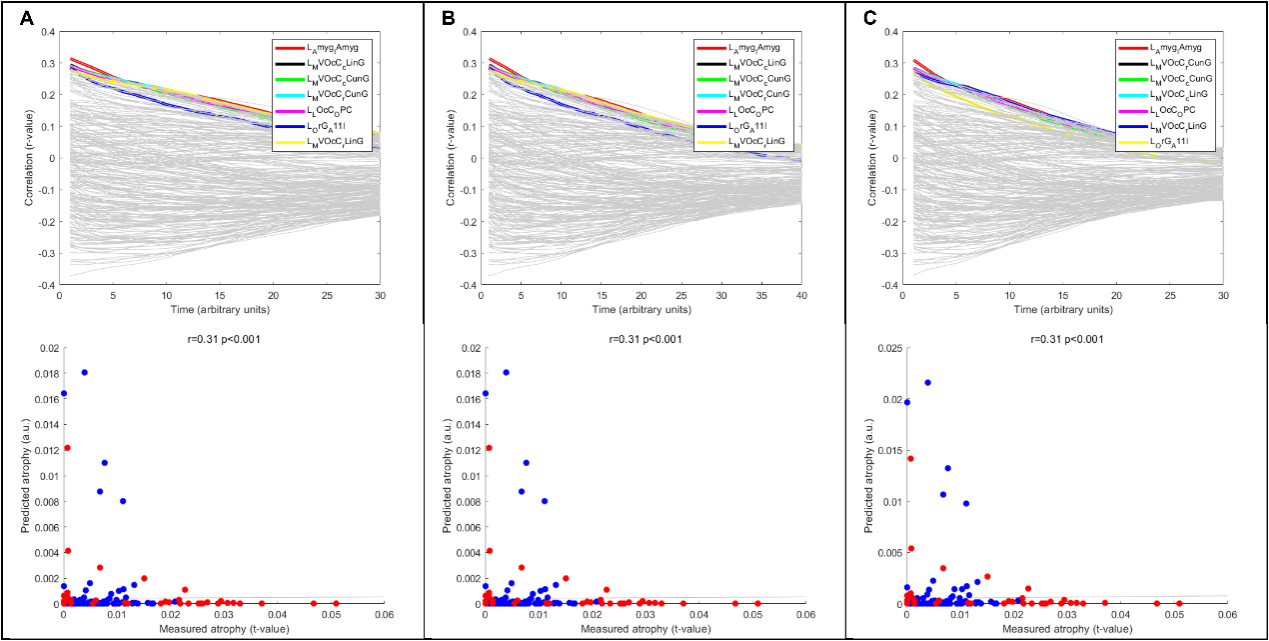


**Figure S 2. Robustness analysis of expansion through NDM.**

**Figure legend:** (A), (B) and (C) show the results of predicting the expansion epicenter of IGD using NDM with different combinations of β and t. (A) β = 0.8, t = 30; (B) β = 0.8, t = 40; (C) β = 1.2, t = 30. The regions showing the highest correlation between the measured and predicted expansion are the left lateral amygdala (red), left cuneus gyrus (blue and green in (A) and (B), black and green in (C)), left caudal and rostral lingual gyrus (black and yellow in (A) and (B), blue and dark blue in (C)), left occipital polar cortex (purple), and left lateral orbital gyrus (dark blue in (A) and (B), yellow in (C)).


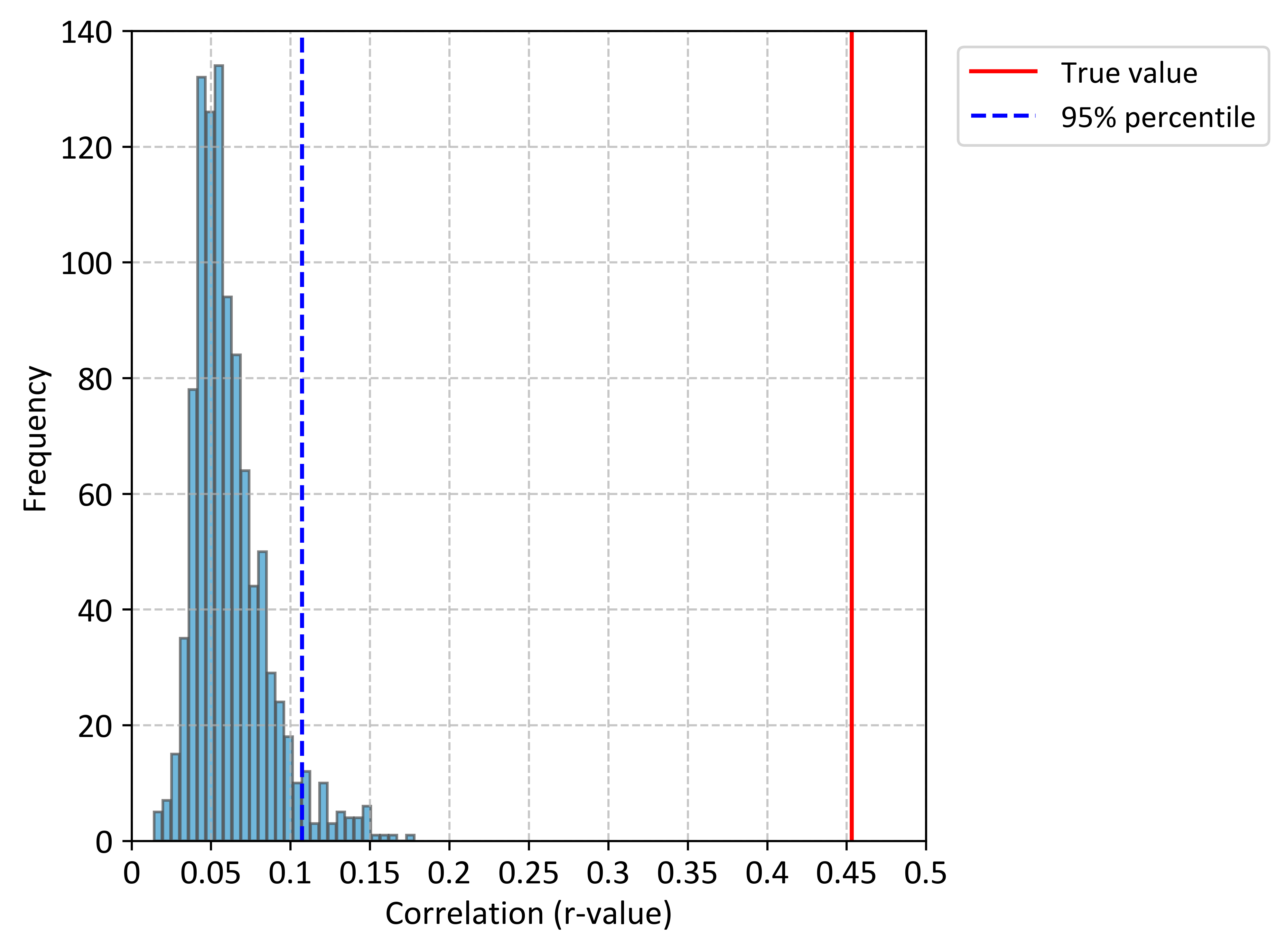


(A)

(B)


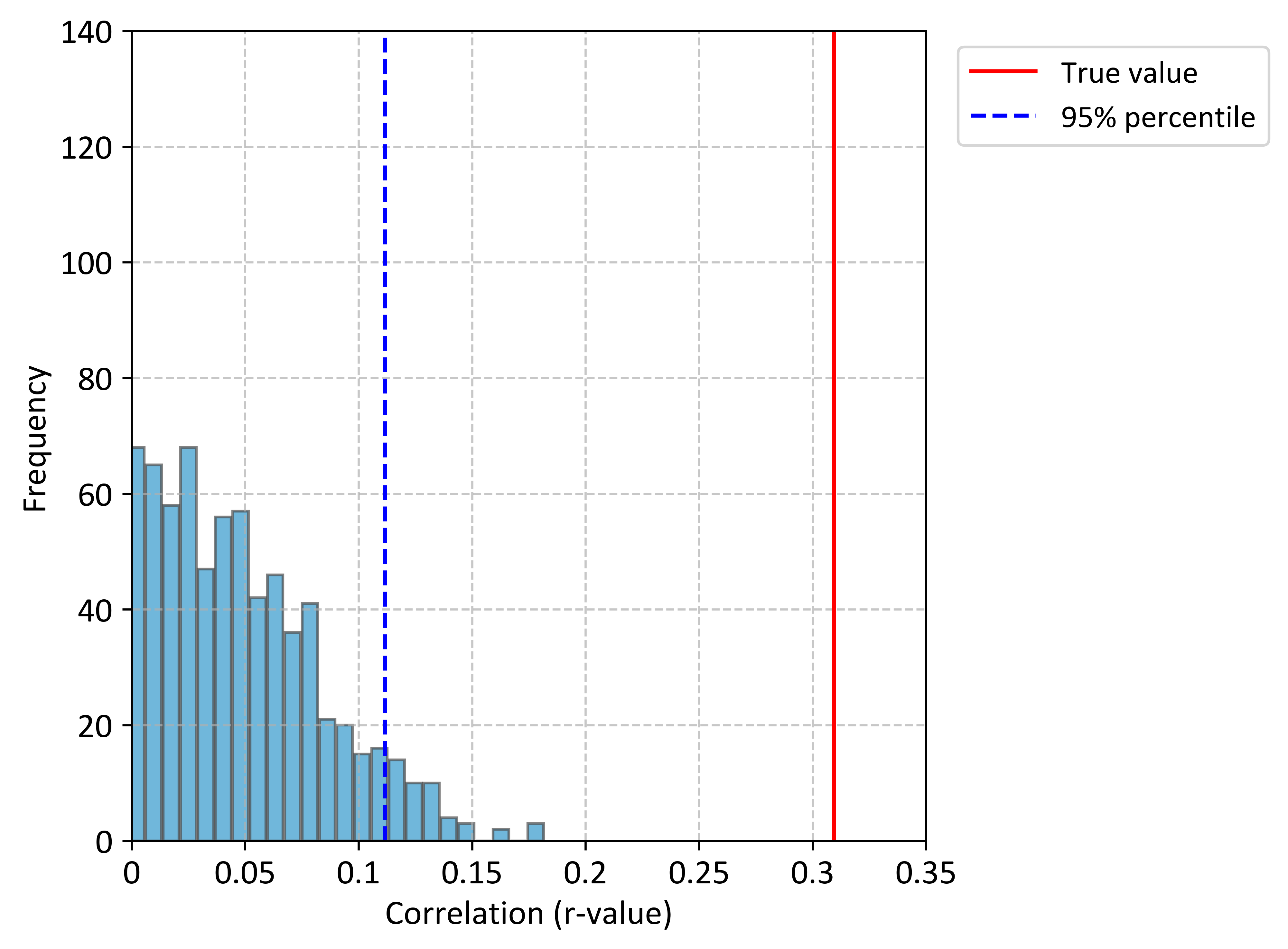


**Figure S 3. Null model validation for the atrophy and expansion epicenters**

(A) Histogram of maximum correlation between predicted and measured GMV atrophy in 1,000 random connectomes preserving degree distribution (right cingulate gyrus as seed). (B) Histogram of maximum correlation between predicted and measured GMV expansion in 1,000 random connectomes preserving degree distribution (left amygdala as seed). The red solid lines indicate empirical correlations (r = 0.453 for atrophy; r = 0.309 for expansion), and blue dashed lines indicate the 95th percentiles (r = 0.107 and 0.112, respectively). Both empirical values significantly exceed the null thresholds (p < 0.001).

The main NDM code, and MINI/DSM-5 assessment forms used in the article can be found at : [Psy-EFGH/Open-access-for-NDM-Predicts-Spatiotemporal-Gray-Matter-Alterations-in-Internet-Gaming-Disorder-: Anonymized GMV matrices, NDM code, and MINI/DSM-5 assessment forms](https://github.com/Psy-EFGH/Open-access-for-NDM-Predicts-Spatiotemporal-Gray-Matter-Alterations-in-Internet-Gaming-Disorder-).
